# A new species of *Austrodecus* Hodgson, 1907 (Arthropoda, Pycnogonida, Austrodecidae) from the Southwest Indian Ridge

**DOI:** 10.3897/zookeys.349.6170

**Published:** 2013-11-13

**Authors:** Jian-Jia Wang, Ding-Yong Huang, Rong-Cheng Lin, Xin-Qing Zheng

**Affiliations:** 1 Third Institute of Oceanography, SOA, Daxue Road No.178, Xiamen 361005, P.R.China

**Keywords:** Southwest Indian Ridge, Pycnogonida, *Austrodecus*

## Abstract

A new species of pycnogonid collected by the Chinese research vessel R/V Dayangyihao during cruises to the Southwest Indian Ridge in 2008 and 2009 is recorded. The new species, *Austrodecus bamberi*, is placed into the *tristanense*-section by the characters of 4-articled ovigers and present auxiliary claws and is distinguished from other species in this section by the number and length of tubercles on the first coxae.

## Introduction

The genus *Austrodecus* Hodgson, 1907 counts 41 named species now (Bamber and El Nagar 2013), predominantly in the southern hemisphere or in the Antarctic and Sub-Antarctic localities. There are four species found in the northern hemisphere, *Austrodecus tubiferum* Stock, 1957 and *Austrodecus palauense* Child, 1983 from western pacific, *Austrodecus conifer* Stock, 1991 and *Austrodecus (Tubidecus) aconaea* (Hedgpeth & McCain, 1971) from the northeastern side of Atlantic and the Pacific respectively. Stock (1957) placed the then known species into four sections based principally on the number of oviger articles and the presence or absence or state of development of the auxiliary claws. He identified these sections as the *glaciale*-section, the *breciceps*-section, the *tristanense*-section and the *gordonae*-section. Bamber and Thurston (1993) published a key to the known species of genus *Austrodecus* not including the species in the *glaciale*-section. Stock (1991) and Child (1994) founded subgenus *Tubidecus* and *Microdecus* based on oviger morphology and the position and shape of the cement gland tube respectively. Child’s (1994) monograph provides the most comprehensive analysis of the genus to date and includs a key to the Antarctic and Sub-Antarctic species. Only two species have since been described: *Austrodecus (Tubidecus) oferrecans* Bamber, 2000 and *Austrodecus childi* Arango, 2003.

Six new hydrothermal fields and two water column hydrothermal anomalies were recorded along Southwest Indian Ridge (SWIR) during Legs 5-7 of the Chinese DY115-20 cruise on the R/V Dayangyihao from 2008 to 2009 (Tao et al. 2009). A series of research studies were undertaken, including benthic surveying, water sampling, and grabbing for biological specimens and geological material along the 48–54°E segment of SWIR. At station DY115-20VII-TVG02, close to the new hydrothermal field (51.732°E, 37.466°S, 1,595m) (Tao et al. 2009), one specimen of Pycnogonida obviously belonged to the genus *Austrodecus* was collected. After checking the known species list, it is conformed as a new species and described below.

## Material and methods

This specimen was collected by a deep sea TV-grab and sorted from the other benthic fauna and sediment from Station DY115-20VII-TVG02. Type material is conserved at the Third Institute of Oceanography, SOA, China. Specimens were drawn using a camera lucida. Measurements are made axially, dorsally for the trunk, laterally for the palp, proboscis and leg.

## Systematics

### Class Pycnogonida Latreille, 1810
Order Pantopoda Gerstäcker, 1863
Suborder Stiripasterida Fry, 1978
Family Austrodecidae Stock, 1954
Genus *Austrodecus* Hodgson, 1907

#### Austrodecus bamberi sp. n.

http://zoobank.org/2DEE9D65-4F7A-487C-8BA6-1EFE8EB1EE7E

http://species-id.net/wiki/Austrodecus_bamberi

Figs 1 , 2

##### Material examined.

one male, holotype (TVG0201), DY115-20VII Station 2, SWI, 37.4654°S, 51.7213°E, 1307 m depth, TVG, 4th February 2009.

**Figure 1.**
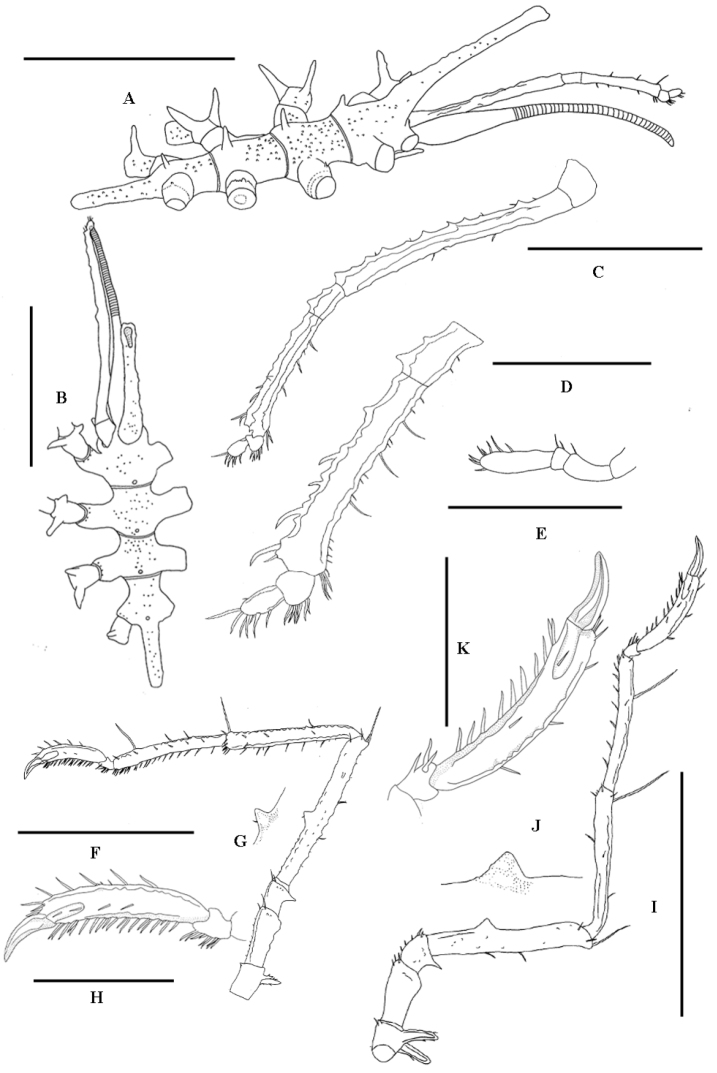
*Austrodec us bamberi* sp. n., TVG0201, male holotype: **A** trunk, lateral view **B** trunk, dorsal view **C** palp **D** terminal articles of palp, enlarged **E** oviger **F** leg 1 **G** cement gland tube of leg 1, enlarged **H** tarsus, propodus, and claws of leg 1, enlarged **I** leg 3 **J** cement gland tube of leg 3, enlarged **K** propodus, and claws of leg 3, enlarged. Scale bars (**A, B, F, I** = 1.0 mm; **C** = 0.5 mm; **D, E, H, K** = 0.25 mm).

##### Diagnosis.

Trunk with four dorsomedian tubercles, moderately tall. All first coxae with slender dorsodistal tubercles, from anterior to posterior in formula 2: 2: 2: 1. The anterior tubercle on the first coxa of first leg is shorter than others. Cement gland dome-shaped, placed dorsally at centre of femur, on all legs. Legs with long dorsodistal spine on each major article.

##### Description of the holotype (male).

Size moderately large for genus; leg span 6.68 mm. Trunk slender, with many tiny papillae, completely segmented, dorsal segmentation lines raised, swollen, with four dorsomedian tubercles, moderately tall. Lateral processes separated by at least their own diameters, with tiny dorsodistal papillae. Ocular tubercle long, directed obliquely forward, slender, armed with short tubercles, without obvious eyes, tip weakly bifurcate. Proboscis typical, slender, longer than trunk length, distal part down-curved, with about 40 annulations, base swollen. Abdomen with tiny dorsal papillae, horizontal, extending to third coxae of fourth pair of legs.

Palps six-articled. First article broad, without spines or setae. Second article longest, with few short seta and spines. Articulation lines between second, third and fourth articles indistinct. Second to fourth articles with thorn-like tubercles on dorsal surfaces. Fourth article almost half length of second article, bearing four inwardly-curved spines, armed with seta. Distal two articles short, terminal article synaxial to penultimate article, both armed with fields of ventral and distal setae mostly little longer than their article diameter.

Ovigers small, four-articled. First to third articles with 1 or 2 setae. Fourth article longest, bearing several ventral and distal setae.

Legs slender, with tiny papillae and thorn-like tubercles. Major articles with long dorsodistal spine. First coxae with tall slender tubercles bearing short thorn-like tubercles and short seta, from anterior to posterior in formula 2: 2: 2: 1. Anterior tubercle on first coxa of first leg shorter than others. Second coxae longest, distally swollen, with short ventral seta and distal spines. Third coxae short, with low tubercle. Femur the longest article, with few short setae and spines. Cement glands dome-shaped, endal at centre of femur, on all legs. Spines on tibiae, tarsus and propodus decreasing in number from anterior to posterior legs. First tibiae longer than second, with fewer ventral spines. Tarsus with ventral spines, 9 on first, 7 on second, 3 on third and fourth pair. Propodus moderately curved, with 3 distal spines and 3-5 long dorsal spines (5 on first, 4 on second, 3 on third and fourth), single row of sole spines (19 on first, 13 on second, 10 on third and fourth). Main claw strong, two tiny auxiliary claws little longer than diameter of main claw.

Female and juvenile are unknown.

*Measurements of holotype in mm*: Trunk length (from chelifore insertion to tip of 4th lateral processes), 1.29; width across 2nd lateral processes, 0.71; proboscis length, 1.35; ocular tubercle, 0.88; abdomen, 0.47.

Length of palp articles 1 to 6 respectively: 0.06;0.73; 0.11; 0.40;0.04;0.06.

Length of oviger articles 1 to 4 respectively: 0.04; 0.07; 0.02; 0.11.

Third leg, coxa 1, 0.15; coxa 2, 0.23; coxa 3, 0.11; femur, 0.67; tibia 1, 0.56; tibia 2, 0.54; tarsus, 0.04; propodus, 0.39; claw, 0.12; auxiliary claw, 0.036.

Measurements of first leg: coxa 1, 0.14; coxa 2, 0.28; coxa 3, 0.19; femur, 0.80; tibia 1, 0.76; tibia 2, 0.65; tarsus, 0.06; propodus, 0.36; claw, 0.12; auxiliary claw, 0.029.

**Figure 2.**
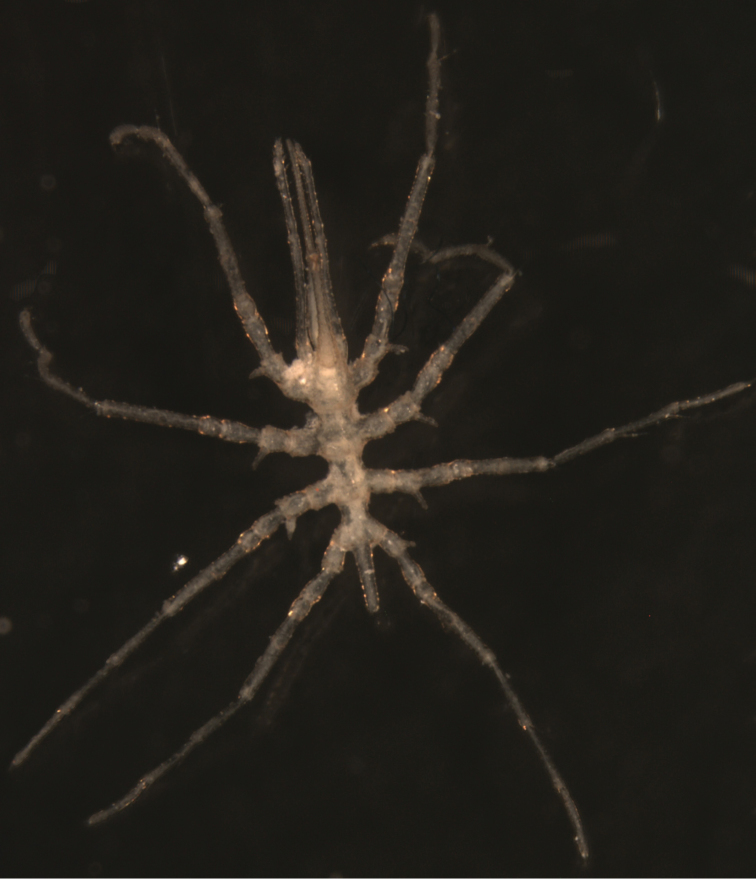
*Austrodecus bamberi* sp. n. male holotype. Photograph from dorsal view.

##### Etymology.

This species is dedicated to Dr. Roger N. Bamber in recognition of his excellent work on the all world’s Pycnogonida.

##### Remarks.

This species belongs to the *Austrodecus tristanense* section *sensu* Stock (1957) which is characterised by 4-articled ovigers and the present of auxiliary claws. Four species are assigned to this section (*Austrodecus tristanense* Stock, 1955, *Austrodecus goughense* Stock, 1957, *Austrodecus elegans* Stock, 1957 and *Austrodecus calvum* Stock, 1991, see Child 1994). Three species possess mid-dorsal trunk tubercles. Of these, *Austrodecus bamberi* sp. n. is most like *Austrodecus calvum* and *Austrodecus elegans* with which it shares the widely-spaced lateral processes however *Austrodecus calvum* does not possess mid-dorsal processes and further differs in the ratio of the lateral tubercles. Using the keys provided by Bamber and Thurston (1993) and Child (1994) *Austrodecus bamberi* keys out to *Austrodecus elegans* but these species are readily distinguished by the number and length of tubercles on first coxae and the much lower mid-dorsal trunk tubercles of *Austrodecus elegans*.

Deep-sea pycnogonids occasionally occurred in the vicinity of hydrothermal vents and deep-sea ridges, however *Sericosura* is the only obligate vent-associated pycnogonid genus (Bamber 2009). *Austrodecus bamberi* was obtained on the top of ridge, close to the hydrothermal field but without evidence any obligate association. White sediment and a small amount of basalt accompanied the specimen in the TV-grab. The specimen was recovered by washing the sediment through a sieve. Corals attached to the basalt, gastropods and one squat lobster (*Heteronida* sp.) were also recovered from the same sample.

The sea floor appears to be predominantly composed of soft sediment.

## Supplementary Material

XML Treatment for Austrodecus bamberi
